# Explaining the Phenomenon of Therapeutic Deadlock in Infertile Women: A Qualitative Phenomenological Study

**DOI:** 10.1155/ogi/7574541

**Published:** 2025-08-08

**Authors:** Somayeh Souri, Behzad Imani, Maryam Maddineshat

**Affiliations:** ^1^Student Research Committee, Hamadan University of Medical Science, Hamadan, Iran; ^2^Department of Operating Room, School of Paramedicine, Hamadan University of Medical Sciences, Hamadan, Iran; ^3^Department of Nursing, Malayer School of Medical Sciences, Hamadan University of Medical Sciences, Hamadan, Iran

**Keywords:** female infertility, qualitative research, treatment failures, women's health

## Abstract

**Background and Objective:** Infertility is an unpredictable condition that presents various physical, psychological, and financial challenges. The uncertainty surrounding the success of treatment options can hinder individuals' ability to cope, potentially leading to adverse outcomes. This study aimed to explore the phenomenon of therapeutic deadlock in women experiencing infertility.

**Method:** This descriptive phenomenological study was conducted from March to November 2024 at infertility centers affiliated with Hamadan University of Medical Sciences in Iran. A purposive sample was used, and 23 semistructured, in-depth, face-to-face interviews were conducted with women who had experienced primary infertility. The collected data were analyzed using the Colaizzi method.

**Findings:** In exploring experiences of infertility, three main themes emerged. (1) Feelings that they were in a last desperate struggle: This theme includes exploring unconventional infertility treatments, seeking treatment with skepticism, and taking risks to achieve the goal. (2) Negative thoughts to overcome a deadlock: This encompasses thoughts of separation and suicidal thoughts. (3) Intentions to resist surrendering to fate: This theme highlights resistance through faith in god against feelings of surrendering to fate.

**Conclusion:** This study examines the phenomenon of therapeutic deadlock among infertile women, highlighting how their cultural and social contexts shape their treatment choices. Despite facing hesitations and potential risks, these women seek treatment in various ways. When confronted with a deadlock in their journey, they often experience negative thoughts and employ different coping strategies, which range from resistance to faith in a higher power and sometimes surrendering to fate.

## 1. Introduction

Infertility presents significant emotional and social challenges for many women. It extends beyond a medical issue, profoundly impacting psychological well-being, interpersonal relationships, and social status [1]. The World Health Organization defines infertility as the inability to conceive after 1 year of unprotected intercourse [2]. Infertility is classified into two types: primary infertility, which refers to the inability to conceive a first child, and secondary infertility, which is the difficulty in conceiving after already having one child. This issue affects over 15% of the global population and is recognized as a critical reproductive health concern [3]. In Iran, the infertility rate has been reported to range between 5.5% and 25% [4].

Culture significantly shapes the meanings attached to infertility. In traditional Iranian culture, a woman's identity is often closely linked to childbearing and motherhood. As a result, women may endure harrowing emotional experiences related to infertility [5]. Infertile women face not only the psychological challenges associated with their condition but also the financial burdens of treatment. They frequently navigate a cycle of hope and despair during treatment, which can lead to frustration and a sense of being deadlocked [6]. Treatment deadlock occurs when long-term treatments fail to yield effective results. This inability to achieve the desired outcomes can lead to significant frustration, causing physical and psychological distress. It can ultimately leave individuals exhausted and unable to persist with treatment [7]. These feelings adversely affect mental health and can severely lower the quality of life for infertile women, impacting their family and social relationships. Women experiencing a treatment deadlock are more susceptible to disorders such as depression, anxiety, and low self-esteem [8]. These emotional struggles can result in social withdrawal, diminished family interactions, and even complete abandonment of the treatment process [9]. Various factors contribute to these experiences, including social pressures, family expectations, high treatment costs, and the complexities of the treatment process. Cultural messages often exacerbate these issues, leading couples to explore a wide range of treatment options, from traditional methods to modern and nontraditional therapies, in search of a solution to their deadlock [10].

A recent study found that nearly 50% of infertile couples in Iran utilize complementary and alternative methods for treatment [11]. Research conducted by Bozorgnezhad and Maleki revealed that infertile women often turn to traditional therapies. The most common methods include prayer therapy, which is used by 21% of participants, and pilgrimage to holy places and vows, each reported by 16% of the women. In contrast, only 2% of cases involved visits to a geriatrician or magic therapy [10]. Latifnejad Roudsari et al. highlighted that both Muslim and Christian women draw support from their religious beliefs and benefit from religious rituals related to infertility [12]. Similarly, a study by Hung et al. found that 96.17% of 8766 infertile women in Taiwan relied on traditional healing methods [13]. Additionally, a survey conducted in Palestine revealed that conventional healers utilize 54 different types of plants to treat infertility, although these plants' toxic and medicinal effects remain unknown [14].

The study of therapeutic deadlock is significant for women experiencing infertility, as it significantly influences their decisions to either continue or abandon treatment. This choice can have a profound impact their mental and physical health [15]. By understanding the phenomenon of therapeutic deadlock, we can identify the behaviors and situations that infertile women encounter when they feel helpless and exhausted. This insight is crucial for developing better interventions to support their health and well-being.

In this context, a qualitative phenomenological approach serves as an effective method for gaining a deeper understanding of the lived experiences of infertile women. By examining individual meanings and interpretations [16], this method allows us to explore the complex dimensions of therapeutic deadlock and its associated consequences. The present study aims to investigate therapeutic deadlock from the perspective of infertile women, thereby providing a deeper understanding of this phenomenon.

## 2. Methods

### 2.1. Study Setting

This qualitative research employed a descriptive phenomenological approach. Husserl's phenomenology philosophy is commonly utilized to understand human experiences as individuals live them. This approach focuses on directly exploring, analyzing, and describing these phenomena to present them as intuitively as possible [17]. The study followed the Consolidated Criteria for Reporting Qualitative Studies (COREQ) [18].

### 2.2. Participants and Sampling

Purposive sampling was utilized to select participants based on specific inclusion criteria in this study, which focused on women experiencing primary infertility. The goal of purposive sampling is to ensure that the sample aligns closely with the study's aims and objectives, thereby enhancing the accuracy and reliability of the data and findings [19]. Before conducting the interviews, the researcher thoroughly explained the purpose of the study and addressed any questions from the participants. After providing all necessary information, consent for audio recording the interviews was obtained. Participants were informed of their right to withdraw from the study without giving a reason. The timing and location of the interviews were arranged based on the participants' preferences. They were conducted in their homes or a quiet, private environment to ensure confidentiality. The sample size was not predetermined; sampling continued until data saturation was reached. A total of 23 participants with primary infertility, recruited from infertility centers affiliated with Hamadan University of Medical Sciences, agreed to participate in the study. Each participant had undergone at least one unsuccessful cycle of assisted reproductive technology treatment. Those participants who had adopted children were excluded from the study.

### 2.3. Data Collection

Data collection began with three unstructured interviews, which were then evaluated to refine the interview process and questions as needed. Following this evaluation, we conducted additional interviews using semistructured questions. All interviews were carried out face-to-face by the primary author. Each interview started with the open-ended question, “Please describe your experiences and feelings regarding infertility and its treatment,” allowing participants to express their experiences in their own words. To facilitate the discussion, probing questions such as “Can you elaborate on that?” or “Could you provide an example?” were used to gather more in-depth information as participants spoke. The duration of the interviews varied from 41 to 90 min. The interviews were recorded with the participants' permission, and the first author transcribed the transcripts verbatim after several listens. The analysis results were shared with the participants to validate the codes extracted from each interview. The selection of the next interviewee was based on the data analysis results from the previous interviews. This sampling continued until data saturation was achieved, meaning no new information or insights were obtained from the interviews.

### 2.4. Data Analysis

The seven-step method proposed by Colaizzi was utilized for data analysis [20]. Initially, the first and second authors (SS and BI) independently and repeatedly read the transcribed interviews to understand the participants' experiences and feelings clearly. Important phrases were selected from the interview texts and summarized. Next, the implicit meanings within these statements were identified and analyzed. The researchers discussed their findings until they reached a consensus, ensuring the interpretations were valid. Themes were then identified and organized into clusters and categories. Clear statements were developed for research themes and subthemes. The findings were presented to the participants to validate the themes and content, and their feedback was provided to the readers to confirm the interpretation and analysis of the data.

### 2.5. Rigor

To ensure the accuracy and robustness of the study's findings, we employed the Guba and Lincoln method, which is based on four criteria: credibility, transferability, dependability, and conformability [21]. We used prolonged engagement, follow-up observations, and samplings with maximum variability to improve data credibility. Other strategies to improve the research's credibility included member checks and involving all authors in the data analysis. We achieved dependability by meticulously transcribing interviews, raw data, and notes. Additionally, two external reviewers assessed the consistency between the raw data and the findings at various stages, incorporating their suggestions and corrections throughout the study. To maintain conformability, the researchers consciously aimed to minimize the impact of their personal opinions during the coding process. The codes were also reviewed by coresearchers, two participants, and an independent researcher with expertise in qualitative methods. A thorough and detailed description of the work steps was provided to ensure transferability and relevance. Participants' quotes were included as they were originally stated. Additionally, the participants' demographic characteristics and the study context were explained in detail, allowing the reader to determine how the study results may apply to their situation.

### 2.6. Ethical Considerations

After receiving the code of ethics (IR.UMSHA.REC.1402.769) from Hamadan University of Medical Sciences, participants were asked for permission to record their conversations during the interviews. They were assured that their information would be kept confidential and that their identities would remain anonymous. After the research, participants received a gift as a token of appreciation for their involvement.

## 3. Results

Twenty-three married women aged 23 to 47 participated in this study (Table 1). Three main themes emerged from the data: “feelings that they were in a last desperate struggle, negative thoughts to overcome a deadlock, and intentions to resist surrendering to fate.” These themes highlighted the phenomenon of therapeutic deadlock as it relates to experiences of infertility (Table 2).

### 3.1. Feelings That They Were in a Last Desperate Struggle

Women facing infertility encounter a range of challenges throughout their treatment journeys. Many women feel intense frustration and helplessness, mainly when their efforts do not produce positive outcomes. In their quest to overcome infertility, they are open to exploring any available methods that may provide a chance of pregnancy despite the uncertainties and potential risks involved.

### 3.2. Exploring Unconventional Infertility Treatments

The experiences of participants show that women experiencing infertility encounter a variety of family, social, and financial challenges. They often endure profound emotional pain, driving them to seek different methods to achieve pregnancy and alleviate their suffering, particularly after facing disappointment with conventional treatments. Influenced by the suggestions of their spouses and those around them, they frequently turn to available and unconventional options for infertility treatment. One participant shared her experience in this journey:

“…I was completely naked when they placed me inside a hot sheepskin filled with medicine. It was very tight and extremely hot inside, making me feel suffocated. They kept insisting that I take some medicine. After a few hours, I began to feel nauseous and was sweating profusely. Exhausted, I repeatedly asked to come out. My grandmother told me I had to stay in the skin longer. I felt increasingly unwell and eventually passed out…” (P2)

### 3.3. Seeking Treatment With Skepticism

A review of participants' experiences revealed that while they did not believe in prayer or those who advocated for it, they were also skeptical about the effectiveness of herbal remedies, considering unconventional methods to be absurd. However, after conventional treatments failed, they turned to these alternative methods out of desperation to improve their condition. One participant shared her experience as follows.

“…My mother-in-law said, let me make you some herbal medicine to help you get pregnant. I didn't take her seriously and thought to myself, if I couldn't get pregnant with IVF, how could I possibly get pregnant with herbal medicine? However, at that moment, I felt like I had no choice, so I said, let me give it a try, even though deep down, I didn't believe it would work and found the idea to be a bit ridiculous…” (P8)

### 3.4. Taking Risks to Achieve Goal

A review of participants' experiences highlighted that women facing infertility often feel frustrated by lengthy treatment processes, high costs, and low success rates. This frustration sometimes drives them to seek alternative methods without fully understanding the potential risks. As a result, this has led to serious complications and, in some instances, worsened their conditions. One participant stated:

“…After we were disappointed with our previous treatment, we decided to consult someone who prescribed herbal medicines to boost our fertility. Both my husband and I took the medicine regularly. My husband also had a sperm test every three months. Unfortunately, after the tests, we discovered that the herbal medicines had worsened the situation, resulting in a loss of four million sperm each cycle…” (P1)

### 3.5. Negative Thoughts to Overcome a Deadlock

Many participants shared their ongoing struggles with infertility. When their efforts to conceive were unsuccessful, they expressed deep despair because their hopes and dreams felt unfulfilled. Some discussed losing their motivation to continue living and even contemplated suicide or separation as a way to escape the pain of infertility.

### 3.6. Thoughts of Separation

Participants shared their frustration regarding previous unsuccessful treatment outcomes and their inability to afford further treatment. Some individuals also discussed the idea of separation, considering it a possible solution to alleviate the pain associated with infertility. They believed that at least one partner might have a better chance at a happier life if they separated. One participant said,

“…I wanted to go to my mother. I told my husband that he should get married. We should separate so that he would be comfortable. I told my husband that I can't continue this anymore… (P5)

### 3.7. Suicidal Thoughts

Some women, due to the exhaustion from infertility and its treatments, experienced a loss of emotional control and had suicidal thoughts. One woman expresses her thoughts this way:

“… I often think about suicide to get relief from my problems. My husband will get married. At least one of us will be relieved. I am tired…” [14].

### 3.8. Intentions to Resist Surrendering to Fate

A review of experiences indicates that women facing infertility often turn to spiritual approaches to cope with their emotions. On the one hand, these spiritual practices can foster resilience through their faith in God. On the other hand, they may lead to a mindset where individuals attribute their inability to conceive to God's will, which can result in giving up on their dreams of becoming parents.

### 3.9. Resistance Through Faith in a Higher Power

The participants in this study believed that God is the creator and owner of the world and that everything occurs by his permission and will. They found that by communicating with God, sharing their limitations, going on pilgrimages, and engaging in specific religious practices, they could resist infertility and maintain hope in their hearts. One participant shared her experience as follows:

“…I hope to have children someday because God's help makes anything possible. Didn't Mary have a husband, and God blessed her with a child? Even though we are both getting older, I still believe in God…” (P6)

### 3.10. Surrender to Fate

Findings indicate that some infertile women, after enduring several unsuccessful treatment cycles, felt as though their prayers and faith in God had gone unanswered. They began to attribute their infertility to God's will, leading them to stop seeking a cure and to accept what they believed was their fate of being childless. One disappointed woman said, “… We are no longer seeking treatment. My husband believes that other women have become pregnant after one encounter. He thinks that if God wills it, we will have children. If we were to have children through treatment, with all the treatment we have undergone, we would have had children already…” (P10).

## 4. Discussion

This study provides important insights into treatment-seeking behaviors and the status of women who are frustrated with infertility treatments.

Infertility significantly impacts multiple aspects of an individual's life and often results in numerous psychological consequences, including anxiety and depression. As a result, individuals experiencing infertility typically seek out a variety of treatment options [22]. This issue becomes even more challenging when infertility treatments fail, leading individuals to explore new and sometimes unconventional approaches. As a result, treatment-seeking behavior patterns are influenced not only by medical and biological factors but also by social, cultural, and psychological elements [10]. The findings of this study suggest that women struggling with infertility often turn to unconventional approaches in addition to conventional treatments. One such method involves placing women in sheepskin soaked in herbal decoctions and asking them to remain there until they become unconscious. A review of the literature shows that various traditional and alternative methods are used around the world to deal with infertility. Common approaches include traditional herbal and animal medicines, acupuncture, energy healing, and religious rituals. These rituals may involve animal sacrifice, chanting, praying, or seeking guidance from sacred sites or spiritual leaders [23]. The findings of this study differ from those of previous research. Recent studies suggest that belief in the effectiveness of alternative methods leads people to use them [24]. Some research indicates that infertile women view complementary and alternative therapies as a means of “healing the body and mind.” However, the relationship between these methods and successful pregnancy outcomes remains unclear [25]. In contrast, our study found that participants approached these alternative methods with skepticism and frustration, often viewing them as ineffective or even ridiculous. Similarly, Australian patients sought alternative methods to enhance fertility despite doubts about their effectiveness [26]. This discrepancy may be due to cultural differences, changing attitudes over time, demographic changes among study samples, and the influence of the media in shaping views and raising awareness.

The results of this study suggest that choosing alternative methods without careful consideration and consideration of possible consequences has, in some cases, led to serious complications and even worsened the condition of some individuals. Some participants reported during interviews that their fertility had worsened after using herbal and folk remedies. Others mentioned nausea, dizziness, and shortness of breath that required hospital treatment and reported prolonged breast swelling as a side effect. In Palestine, herbalists use a variety of drugs for which there is no scientific evidence of their pharmacological or toxicological effects in the treatment of male and female infertility [14]. Nevertheless, these methods are often considered safe, although their effectiveness remains uncertain and may negatively affect fertility [27]. In addition, other studies have shown limited evidence of the efficacy of complementary and alternative therapies for enhancing fertility [28]. The variability in results regarding the effectiveness of these treatments may be due to differences in the quality and composition of herbal medicines and individual differences such as health status, severity of infertility, and lifestyle.

The use of methods with unknown effects is of concern and can lead to complications, including life-threatening situations. Furthermore, many infertile women are hesitant to disclose their use of these products to health professionals [29]. Therefore, healthcare providers should pay more attention to the use of these medicines among women experiencing infertility. More research is needed to confirm the effectiveness of these treatments.

Our findings indicate that the challenging circumstances surrounding infertility and its treatment can have significant emotional and psychological impacts, straining the couple's relationship. These experiences can cause individuals to question the meaning of life and create uncertainty about the future. Participants reported that they had considered suicide in times of despair and exhaustion. One even reported attempting suicide. Research suggests that approximately 9.4% of individuals may be at risk of suicide due to these challenges. Women who have attempted suicide have shown higher levels of depression and are more likely to be childless or have fewer children [30]. Furthermore, a recent study found that the infertility treatment process can produce a variety of psychological consequences, including difficulties in controlling behavior, feelings of hopelessness and exhaustion, negative thoughts, sadness, and depression [8]. While the same participant was able to manage his negative emotions with the help of counseling programs after a failed suicide attempt, it is clear that counseling, both during treatment and after failures, can significantly enhance mental health and improve quality of life.

Some participants in this study reported that they had proposed separation to their partners out of desperation and a desire to resolve what seemed to be a deadlock, in the hope that at least one partner might have a child. A Swedish study found that half of the participants had separated from their partners, and in all cases, the men had left the women [31]. Furthermore, a recent study found that threats to marital stability were more often initiated by husbands, who sometimes expressed a desire to remarry [8]. These findings contrast with previous research that focused on separations initiated by husbands as a consequence of infertility. Proposing separation by female partners can be driven by a variety of factors, including low economic status, financial dependence on male partners, uncertainty about treatment options, and lack of social and family support. Therefore, providing health counseling and allocating government funds to cover treatment costs could help couples manage the stress of infertility.

The findings of this study suggest that religious and spiritual experiences have dual aspects. On the one hand, participants were able to resist the stress of infertility and maintain hope through their spiritual experiences. An Iranian study confirms this, showing that spiritual experiences help reduce feelings of failure and relieve infertility-related stress. The study also suggests that spiritual experiences act as a form of adaptation and problem-solving, enabling individuals to achieve personal harmony, internal and external integration, and effectively navigate life crises [32]. Research by Kasu et al. showed that women often turn to spirituality as a way to manage infertility-related stress, enabling them to reframe their experiences and find meaning in them [33]. The consistency of these results can be attributed to the universality of religious and spiritual beliefs, which act as a common mechanism for coping with stress across different cultures. Spirituality and religious practices, such as prayer, help reduce stress, reshape experiences meaningfully, and foster a sense of peace. The prominent role of religion in various cultures naturally encourages individuals to draw upon these resources. Additionally, religious social structures provide essential support and hope in facing challenges like infertility.

Other findings from this study suggest that religious and spiritual beliefs can hurt individuals. Women who experience infertility and become frustrated with their treatment often feel that their prayers and trust in God are going unanswered. As a result, they begin to accept infertility as a predetermined fate determined by God's will and may eventually stop seeking treatment. Research suggests that a fatalistic view among infertile women can lead to negative emotions such as anger, hopelessness, feelings of abandonment, and feelings of being ignored by God [34]. Some studies have also shown that dissatisfaction with God is often temporary and occasional. After a short period, many participants seek forgiveness and return to their faith [12].

Different cultural and religious backgrounds can influence how people interpret and accept fatalistic beliefs and receive treatment. While avoiding stressful situations related to fertility can help reduce treatment-related distress in the short term, overreliance on these strategies can have negative consequences [35]. These complex emotions are not surprising after enduring challenging treatments and repeated failures. However, the decision to discontinue treatment due to a fatalistic view should be carefully considered by healthcare professionals and religious counselors.

## 5. Limitations

The main limitation we encountered in this study was the difficulty of sampling. Despite the high prevalence of infertility in Iran, many infertile women were reluctant to participate in the study, and building trust with them required considerable effort. Although we could include participants from various backgrounds and maintain contact with them throughout the study, we did not include their spouses. To gain a comprehensive understanding of infertility and its treatment, it is essential to consider the experiences of both women and men.

## 6. Conclusions

The results of this study indicate that infertile women often seek solutions to their infertility based on their cultural and social circumstances, particularly during times when conventional treatments are ineffective. Despite their uncertainties and the potential risks, they may explore unconventional methods in their search for a solution. When faced with a deadlock, infertile women commonly experience negative thoughts and adopt various strategies to cope with the associated stress. Some women continue to maintain their faith in a higher power and remain hopeful, while others come to accept their childlessness and decide to cease further treatment.

## 7. Recommendations for Future Studies

This study suggests that future research should focus on the behaviors of infertile individuals, especially in the context of treatment failure. Examining the mental barriers that may prevent individuals from seeking medical attention is essential. In addition, the effects of traditional and alternative methods women use on their health could be examined. Given the lack of awareness about these methods among infertile women, health professionals should be aware of the unconventional treatments that these women may be pursuing. In addition, women should receive clear information about the possible side effects of these alternative methods.

## Figures and Tables

**Table 1:** Sociodemographic and clinical characteristics of study participants.

Participants	Age	Infertility duration (years)	Occupation	Educational level	Cause of infertility	Type of treatment
P1	36	14	Housewife	Diploma	Both	IVF, ZIFT, IUI
P2	33	6	Housewife	Bachelor's degree	Both	IVF
P3	44	13	Employee	Bachelor's degree	Female	IUI, IVF
P4	35	8	Nurse	Bachelor's degree	Female	IVF, laparoscopy
P5	37	9	Trader	Primary	Female	IUI, IVF
P6	43	18	Housewife	Diploma	Male	IVF
P7	38	18	Hairdresser	Diploma	Male	IVF
P8	37	11	Housewife	Diploma	Both	IVF
P9	41	10	Teacher	Bachelor's degree	Female	IVF, laparoscopy
P10	47	25	Farmer	Primary	Female	IVF
P11	23	3	Housewife	Diploma	Both	IVF
P12	39	17	Tailor	Primary	Female	IVF
P13	45	12	Housewife	Primary	Both	IUI, IVF
P14	34	4	Housewife	Bachelor's degree	Both	IUI, IVF
P15	36	5	Housewife	Bachelor's degree	Both	IUI, IVF
P16	35	6	Housewife	Diploma	Both	IVF
P17	42	5	Employee	Bachelor's degree	Female	IVF
P18	23	3	Hairdresser	Diploma	Female	IUI, IVF
P19	35	10	Teacher	Bachelor's degree	Female	IVF
P20	44	9	Housewife	Bachelor's degree	Both	IVF
P21	34	8	Nurse	Bachelor's degree	Female	IUI, IVF
P22	39	14	Tailor	Diploma	Both	IVF
P23	45	13	Stockman	Diploma	Male	IVF

**Table 2:** The themes and subthemes.

Themes	Subthemes
Feelings that they were in a last desperate struggle	Exploring unconventional infertility treatments
	Seeking treatment with skepticism
	Taking risks to achieve goal

Negative thoughts to overcome a deadlock	Thoughts of separation
	Suicidal thoughts

Intentions to resist surrendering to fate	Resistance through faith in a higher power
	Surrender to fate

## Data Availability

The data that support the findings of this study are available from the corresponding author upon reasonable request.
